# Adaptive function and correlates of anxiety during a pandemic

**DOI:** 10.1093/emph/eoab037

**Published:** 2021-11-12

**Authors:** Gul Deniz Salali, Mete Sefa Uysal, Abi Bevan

**Affiliations:** 1 Department of Anthropology, University College London, London WC1H 0BW, UK; 2 Psychology Department, Dokuz Eylül University, Izmir, Turkey; 3 Department of Social Psychology, Friedrich-Schiller-Universität Jena, Jena, Germany

**Keywords:** anxiety, evolution, intolerance of uncertainty, time perspective, risk avoidance, COVID-19

## Abstract

**Background and objectives:**

Most studies to date have focused on the negative aspects of anxiety. Anxiety, however, is an evolved emotional response that can provide protection in the face of risk. Pandemics are characterized by increased mortality risk coupled with future uncertainties, which both cause heightened anxiety. Here, we examine the factors associated with anxiety levels and risk avoidance behaviours during the first wave of the COVID-19 pandemic. We asked how individual time perspectives (future-oriented consideration and attention to present moment experience) affect anxiety in uncertain times, and whether anxiety reduces mortality risk by promoting risk avoidance behaviour.

**Methodology:**

We conducted an online survey in the UK (*N* = 1088) and Turkey (*N* = 3935) and measured participants’ generalized and pandemic-related anxiety levels, future-oriented consideration, mindfulness, intolerance of uncertainty, risk perception and risk avoidance behaviours.

**Results:**

We found that people less tolerant of uncertainties had higher levels of pandemic anxiety. Those with higher pandemic anxiety exhibited risk avoidance behaviours more frequently. Mindfulness and increased financial satisfaction reduced pandemic anxiety. People in Turkey reported higher levels of generalized and pandemic anxiety and greater engagement in risk avoidance behaviours than people in the UK.

**Conclusions and implications:**

Our study shows an elevated anxiety response can help mitigate infection risk during pandemics and emphasizes the importance of the underlying situation in understanding whether an anxiety response is adaptive or pathological. Maintaining a healthy level of anxiety can promote engagement in protective behaviours. Therapies addressing anxiety can focus on increasing tolerance to future uncertainties.

**Lay summary:**

Anxiety is an emotional response triggered in the anticipation of a possible threat. We found that intolerance of uncertainty strongly predicted anxiety and that people with elevated anxiety levels engaged in protective behaviours more frequently during the COVID-19 pandemic, suggesting that anxiety can help mitigate mortality risk.

## INTRODUCTION

Anxiety is an emotional response triggered in the anticipation of a possible threat. From an evolutionary perspective, anxiety can be seen as a detector that helps an individual to prepare for and deal with a dangerous situation [1, 2]. Pandemics like the COVID-19 are characterized by high level of threat, i.e. risk of infection and mortality, coupled with future uncertainties. These characteristics are expected to result in an increase in anxiety responses across populations. Nevertheless, how people cope with uncertainties will depend on individual-level traits such as how much a person is invested in their future. Being too future-oriented may come at a cost because constantly planning and reflecting about the future may increase individuals’ anxiety levels, especially at times with high future uncertainties. On the other hand, elevated levels of anxiety during a pandemic can be part of an adaptive response that has evolved to minimize mortality risk. In this article, we examine the factors associated with anxiety levels during the first wave of the COVID-19 pandemic in the UK and Turkey, focusing on individual time perspectives, and ask whether anxiety has an adaptive role in times of pandemics.

### Future orientation: a potential mismatch leading to anxiety?

Because anxiety is an emotional response that occurs in the anticipation of a threat in the future, how much an individual considers future outcomes, and their degree of future orientation may affect their anxiety levels. While there is much individual variation in future orientation [3], many people living in Western countries often think and plan for long-term futures [4]. In modern societies, future-oriented plans are vast: investment accounts, pension benefits, insurance schemes, etc. Future orientation, however, would have been less useful during most of human evolution. Research on contemporary hunter-gatherers in Congo has shown that forest hunter-gatherers discount the future more than neighbouring farmers and hunter-gatherers who are more market-integrated [5]. This suggests that future orientation in humans is a flexible behavioural adaptation associated with the emergence of food storage systems and agriculture [5]. Future orientation in modern societies is at the extreme end of the time perspective spectrum, where the amount of time needed to achieve many personal goals is counted in years. This is very much in mismatch with the duration of goals set by a prehistoric hunter-gatherer who consumed food immediately, did not store and accumulate materials, and ‘lived in the present’. An evolutionary perspective on happiness predicts that the size and duration of personal goals in modern societies may be the major contributor to the current mental health problems [6]. Following this perspective, the observed mismatch in time perspectives may be contributing to the increased levels of anxiety and mood disorders in recent years [7].

Previous studies on the link between future orientation and anxiety have shown mixed results. In one study, trait anxiety was associated with less future discounting, i.e. more future orientation [8]. Two studies found a weak but significant negative relationship between future orientation and anxiety [3, 9]. However, as the authors of one of those studies acknowledged the negative association may be due to the future scale used in the studies (Zimbardo Time Perspective Inventory) that focused on measuring the expectations of a positive future and rewards [9]. Nevertheless, attribution of negative outcomes to future events is at the core of an anxiety response [10]. Studies have shown that future *negative* time perspectives are significantly associated with anxiety [11, 12], and the majority of worry contents concern the uncertain future [13]. Indeed, worriers often interpret ambiguous situations as threatening and *intolerance of uncertainty* is strongly associated with anxiety [14, 15]. Because worry occurs as a mental problem-solving in response to anticipation of negative future events [16], we predict that the combination of too much future-orientation (a feature of modernity) and a tendency towards attributing negative outcomes to future uncertainties will be positively associated with anxiety levels during a pandemic, when future uncertainties prevail.

Following on the predicted association between future orientation and anxiety, we can expect that attention to present moment may reduce anxiety by taking one’s focus away from potential future outcomes, including during pandemics. A relevant concept here is mindfulness which is defined as the awareness of and attention to experiences in the present moment [17]. Mindfulness is associated with a reduced focused on the negative aspects of the past and negative predictions of the future [18] and predicts positive affect [19]. In this article, we include mindfulness, in addition to future orientation, as a time perspective covariate and predict it to be negatively correlated with pandemic anxiety.

### Adaptive function of anxiety: signal detection

Although much research on trait anxiety has focused on its negative effects and therapeutic solutions, it is important to acknowledge that emotions serve a purpose. They are systems of response shaped by natural selection in response to threat or opportunity situations [20, 21]. Anxiety, for example, prepares the individual to detect and handle threats [1, 2]. Because there is often ambiguity in whether a threat is present and absent, how is the threat response optimised? Natural selection shapes regulation mechanisms according to the principles of signal detection theory. Individuals vary in the threshold above which they accept the evidence that the threat (or any event) is present [1]. One prediction from this theory is that in an environment where there are many threats, the threshold for threat detection should be lower, leading individuals to present more anxiety symptoms [2, 21]. Moreover, intrinsic individual variation in the threshold for threat detection results in variation in susceptibility to anxiety. Those with lower thresholds for threat detection experience higher levels of anxiety [2].

The optimal response threshold depends on the costs and benefits of expressing the defence response. Expressing a false alarm when there is no predator in the jungle can cost a forager a few calories that they could be obtaining. Nevertheless, not firing an alarm when there is a predator can be much more costly (death). That is why according to the ‘smoke detector principle’ many more false alarms are expected in an optimal defence response [22]. Anxiety is one such defence response, benefiting individual survival and reproduction by decreasing the risk of mortality, and is expected to be ‘fired falsely’ in certain situations [23]. Adolescents with higher levels of trait anxiety, for example, are found to have reduced risk of mortality from accidents in later life [24]. Anxiety comorbid with depression was found to reduce mortality compared with depression alone in Norwegian adults [25]. In a recent study, we showed that anxiety levels were positively associated with accepting a COVID-19 vaccination [26].

Because pandemics are situations where mortality risk is elevated, we expect to observe an overall heightened anxiety levels during a pandemic. Moreover, following the application of the signal detection theory to anxiety disorders [2], and earlier empirical studies [27], we predict that individuals with lower thresholds for exhibiting a threat response, i.e. those with elevated levels of risk perception, will have increased anxiety levels during a pandemic. Because anxiety is a defence response against potential threats to survival, we also predict that those with increased anxiety will engage in risk avoidance behaviours, such as complying with social distancing measures or staying at home, more frequently.

To test the above predictions on the correlates and potential adaptive function of anxiety during a pandemic, we conducted an online survey in the UK and Turkey in April and May 2020, when both countries were going through the first wave of the COVID-19 pandemic.

We hypothesized:

- The overall anxiety level of a person (i.e. generalized anxiety) will be positively associated with their level of pandemic-related anxiety.
- Future orientation and intolerance of uncertainties will be positively and mindfulness will be negatively associated with pandemic anxiety.
- Perceived risk of catching the novel coronavirus will be positively associated with pandemic anxiety.
- Participants with increased levels of pandemic anxiety will engage in risk avoidance behaviour more frequently.

We conducted a study in the UK and Turkey to examine whether the above hypotheses will be supported across different cultures. We also controlled for demographic variables that may be correlated to the anxiety response. These included age, sex, education and financial satisfaction.

## METHODOLOGY

### Participants

We distributed the link to the online survey through social media (Twitter and Facebook), email and WhatsApp groups. Posts briefly explained the purpose of the study (‘with this anonymous survey, we hope to understand the emotional and behavioural response against the pandemic and future uncertainty in the UK and Turkey comparatively’) and requested those over 18 and living in the UK and Turkey participate. Hashtags such as ‘#pandemic’ ‘#covid19’ and ‘#research’ were used to reach people searching for those terms on relevant platforms. The link to the survey was also shared on social media pages of popular science platforms that shared studies on COVID-19 at the time. Participation was voluntary and anonymous and did not involve any compensation. Informed consent was obtained from all participants. A bilingual website was set up to provide information about the study and link to the survey and to share early results with those who had participated and with the wider public. The study flyer that was used to recruit participants online can be found at https://uclanthrosurvey.wixsite.com/covid19/home.

Data were collected during the first wave of COVID-19 pandemic in April and May 2020 (from 27 April 2020 to 25 May 2020), when both countries were in national lockdown. A total 6067 self-identified Turkish participants and 1534 self-identified UK participants participated in the study. We excluded the participants who did not complete the survey until the end and who did not live in Turkey and UK. The final sample was composed of 5023 participants (3935 Turkish and 1088 UK). Supplementary Table S1 lists demographic information of the study participants in each country. The study was approved by the UCL Research Ethics Committee (ethics ID: 13121/002), and the methods were carried out in accordance with the approved guidelines.

### Study variables

Table 1 lists all the study variables, along with the corresponding survey questions, response scales and summary statistics of the measured variables in each country. We measured overall anxiety levels using the seven-item generalised-anxiety disorder assessment, GAD-7 [28]. For pandemic-related anxiety levels, we generated a six-item questionnaire related to the worries a person may be experiencing during the COVID-19 pandemic (*α* = 0.77 for both countries). To assess participants’ risk avoidance behaviour during the pandemic, we generated a six-item questionnaire (*α* = 0.87 for UK and *α* = 0.80 for Turkey). We measured future orientation by using six items of the two-factor Consideration of Future Consequences Scale, CFC-14 (*α* = 0.77 for UK and *α* = 0.70 for Turkey) [29]. To measure uncertainty intolerance, we used three items of Intolerance of Uncertainty Scale, IUS-12 (*α* = 0.70 for UK and *α* = 0.72 for Turkey) [30, 31]. We used five items of Mindful Attention Awareness Scale (MAAS) to measure mindfulness (*α* = 0.80 for UK and *α* = 0.66 for Turkey) [19]. We measured risk perception by asking participants about their perceived risk of catching the novel coronavirus. When the original scales were shortened, we did so by the relevance of the scale item to our study purpose and the corresponding factor loadings in previous studies. Finally, we controlled for age, sex, education and financial satisfaction (Supplementary Table S1).

**Table 1.** Variables used in the study

Variable name	Statement	Response scale	UK, *M* (*SD*) or *n* (%)	Turkey, *M* (*SD*) or *n* (%)
Generalized anxiety	Over the last 2 weeks, how often have you been bothered by any of the following problems?	1 = not at all, 2 = several days, 3 = more than half the days, 4 = nearly every day	1.86 (0.73)	2.14 (0.72)
GAD-1	Feeling nervous, anxious or on edge		2.05 (0.97)	2.41 (0.93)
GAD-2	Not being able to stop or control worrying		1.72 (0.90)	1.94 (0.95)
GAD-3	Worrying too much about different things		1.99 (0.97)	2.28 (0.96)
GAD-4	Trouble relaxing		1.95 (0.95)	2.25 (0.96)
GAD-5	Being so restless that it is hard to sit still		1.64 (0.88)	1.77 (0.86)
GAD-6	Becoming easily annoyed or irritable		1.99 (0.89)	2.40 (0.97)
GAD-7	Feeling afraid as if something awful might happen		1.68 (0.87)	1.90 (0.91)
COVID-19 (pandemic)-related anxiety	To which extent do the following statements apply to you right now?	1 = does not apply at all, 2 = applies a little, 3 = somewhat applies, 4 = applies very much	2.36 (0.68)	2.89 (0.67)
PRA-1	I am worried about the health of my family member(s) and/or friends		3.00 (0.91)	3.35 (0.83)
PRA-2	I am worried about my health		2.21 (0.94)	2.50 (0.98)
PRA-3	I am worried about losing my job or experiencing financial loss		2.07 (1.08)	2.89 (1.10)
PRA-4	I am worried about passing coronavirus on to others		2.54 (0.99)	3.13 (1.02)
PRA-5	I am feeling anxious and fearful		2.19 (1.01)	2.52 (0.99)
PRA-6	I feel stressed about leaving my house		2.12 (1.04)	2.98 (0.99)
Risk avoidance behaviours	To what extent do the following statements describe your behaviour at the START (i.e. the first confirmed case/death) of the COVID-19 epidemic in your country?	1 = does not apply at all, 2 = applies a little, 3 = somewhat applies, 4 = applies very much	2.56 (0.85)	3.46 (0.60)
RAB-1	I stopped attending social gatherings		2.85 (1.22)	3.65 (0.72)
RAB-2	I kept at a distance of at least two meters (six feet) to other people		2.62 (1.22)	3.25 (0.87)
RAB-3	I stayed at home		2.48 (1.18)	3.39 (0.88)
RAB-4	I washed my hands frequently		3.31 (0.92)	3.73 (0.58)
RAB-5	I wore a mask when I went outside		1.26 (0.70)	3.10 (1.17)
RAB-6	I avoided crowded places		2.83 (1.16)	3.64 (0.70)
Future-oriented consideration	For each of the statements below, please indicate whether or not the statement is characteristic of you.	1 = extremely uncharacteristic (not at all like you), 2 = somewhat uncharacteristic, 3 = uncertain, 4 = somewhat characteristic, 5 = extremely characteristic (very much like you)	3.61 (0.77)	3.42 (0.65)
FC-1	I only act to satisfy immediate concerns, figuring the future will take care of itself.(*Reverse Coding*)		3.64 (1.22)	3.47 (1.12)
FC-2	My behaviour is only influenced by the immediate (i.e., a matter of days or weeks) outcomes of my actions. (*Reverse Coding*)		3.61 (1.16)	3.02 (1.04)
FC-3	I only act to satisfy immediate concerns, figuring that I will take care of future problems that may occur at a later date. (*Reverse Coding*)		3.45 (1.23)	3.37 (1.10)
FC-4	Often I engage in a particular behaviour in order to achieve outcomes that may not result for many years.		3.16 (1.15)	3.30 (1.05)
FC-5	I am willing to sacrifice my immediate happiness or well-being in order to achieve future outcomes.		3.66 (1.03)	3.48 (0.98)
FC-6	When I make a decision, I think about how it might affect me in the future.		4.13 (0.90)	3.89 (0.90)
Intolerance of uncertainty scale	For each of the statements below, please indicate whether or not the statement is characteristic of you.	1 = extremely uncharacteristic (not at all like you), 2 = somewhat uncharacteristic, 3 = uncertain, 4 = somewhat characteristic, 5 = extremely characteristic (very much like you)	2.94 (1.01)	3.49 (0.93)
IUS-1	My mind can’t be relaxed if I don’t know what will happen tomorrow.		2.84 (1.31)	3.34 (1.22)
IUS-2	Uncertainty makes me uneasy, anxious, or stressed.		3.50 (1.26)	4.00 (1.02)
IUS-3	When it’s time to act, uncertainty paralyses me.		2.49 (1.28)	3.13 (1.22)
Mindful attention awareness scale	Please indicate how frequently or infrequently you currently have each experience	1 = almost never, 2 = very infrequently, 3 = somewhat infrequently, 4 = somewhat frequently, 5 = very frequently, 6 = almost always	3.85 (0.92)	3.51 (0.85)
MAAS-1	I rush through activities without being really attentive to them. (*Reverse Coding*)		4.02 (1.18)	3.97 (1.33)
MAAS-2	It seems I am ‘running on automatic’, without much awareness of what I’m doing. (*Reverse Coding*)		3.94 (1.27)	3.65 (1.36)
MAAS-3	I find myself preoccupied with the future or the past. (*Reverse Coding*)		3.30 (1.36)	4.06 (1.33)
MAAS-4	I get so focused on the goal I want to achieve that I lose touch with what I’m doing right now to get there. (*Reverse Coding*)		4.23 (1.18)	3.29 (1.35)
MAAS-5	I find myself doing things without paying attention. (*Reverse Coding*)		3.78 (1.20)	2.60 (1.20)
Risk perception	If you haven’t been tested positive for COVID-19 or did not show COVID-19 symptoms, what do you think is the probability of you catching the coronavirus?	‘0’ means there is no chance you think you will catch coronavirus, and ‘100’ means you will definite	50.67 (23.50)	49.77 (25.86)

### Statistical analysis

We first examined bivariate relationships across all variables for each country. Table 2 shows the correlations among the theoretically important variables. We then conducted multiple linear regression analyses, for each country, to examine the predictive power of each of the variables on (i) pandemic-related anxiety levels and (ii) risk avoidance behaviours during the pandemic. We examined the country-level differences in the mean generalized and pandemic-related anxiety levels, intolerance of uncertainty, mindful attention awareness and risk avoidance behaviour scores using pairwise *t*-tests. Statistical analyses were conducted using SPSS (version 25) and R (version 4.0.3). Datafiles and the R code are available at OSF (https://osf.io/9wu2f/).

**Table 2.** Correlations among variables

Variables	RAB	PRA	GAD	FC	IUS	MAAS	RP	FS
Risk avoidance behaviours (RAB)	–	0.22^***^	0.11^***^	−0.02	−0.03	0.04	0.05	−0.10^**^
Pandemic-related anxiety (PRA)	0.21^***^	–	0.64^***^	0.04	0.43^***^	−0.40^***^	0.20^***^	−0.33^***^
Generalized anxiety (GAD)	0.04^*^	0.51^***^	–	0.05	0.54^***^	−0.48^***^	0.12^***^	−0.31^***^
Future-oriented consideration (FC)	0.06^***^	0.12^***^	0.07^***^	–	0.10^**^	0.01	0.03	0.09^**^
Intolerance of uncertainty (IUS)	0.01	0.43^***^	0.54^***^	0.11^***^	–	−0.53^***^	0.13^***^	−0.20^***^
Mindfulness Attention Awareness Scale (MAAS)	0.00	−0.29^***^	−0.44^***^	0.02	−0.43^***^	–	−0.09^**^	0.23^***^
Risk perception (RP)	0.00	0.22^***^	0.14^***^	0.05^**^	0.09^***^	−0.08^***^	–	−0.03
Financial satisfaction (FS)	−0.04^*^	−0.22^***^	−0.23^***^	0.07^***^	−0.14^***^	0.13^***^	−0.03^*^	–

*Note.* The upper right of the diagonal displays results for the UK, and the lower left of the diagonal displays results for Turkey.

* *P* < 0.05,

** *P* < 0.01,

*** *P* < 0.001.

## RESULTS

### Generalized anxiety was highly correlated with pandemic anxiety

**Figure 1.**
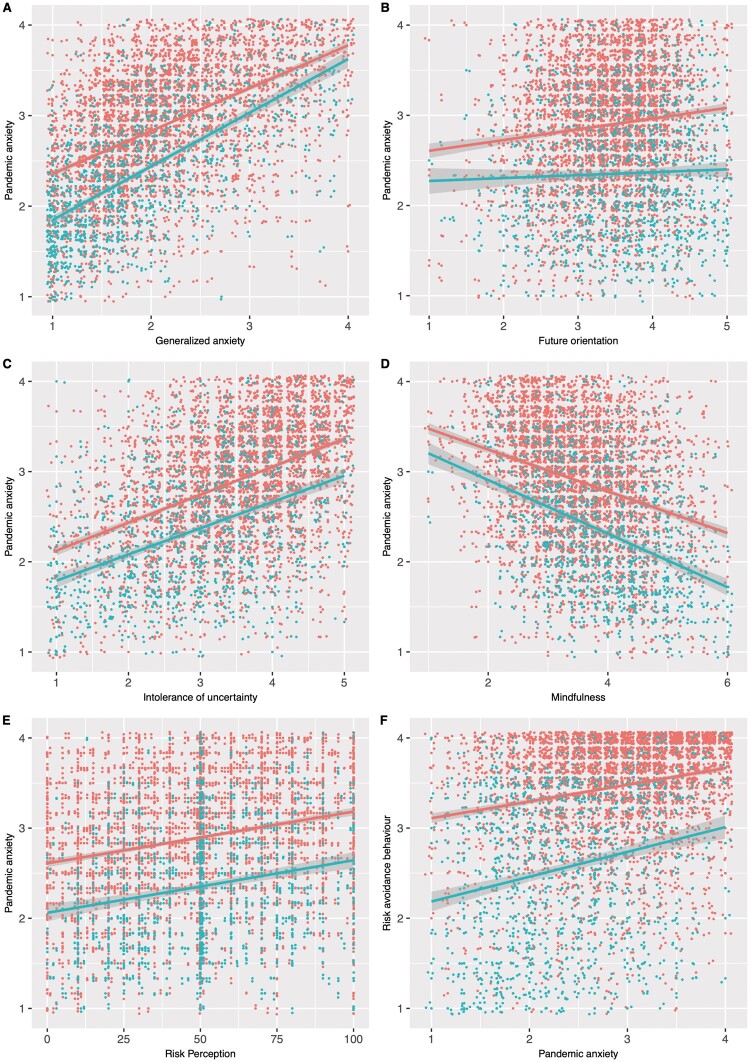
The correlation of pandemic anxiety with (**A**) generalized anxiety, (**B**) future-oriented consideration, (**C**) intolerance of uncertainty, (**D**) mindful attention awareness, (**E**) perceived risk of catching the virus and (**F**) risk avoidance behaviour. Red are datapoints from Turkey and blue are from the UK. We used jittered points to visualize overlapping datapoints

In line with the prediction from our hypothesis 1, generalized anxiety scores were strongly correlated with pandemic-related anxiety scores in both countries (for UK: *B* = 0.64, *P* < 0.001, for Turkey: *B* = 0.51, *P* < 0.001). People who were more anxious in general had also increased pandemic-related anxiety (Fig. 1A).

### Intolerance of uncertainty was the strongest predictor of pandemic anxiety in both UK and Turkey

We predicted future orientation and intolerance of uncertainty to be positively associated with pandemic anxiety (hypothesis 2). In line with this hypothesis, future-oriented consideration scores predicted pandemic anxiety; however, only in Turkey, this association was significant, and the effect sizes were small (Table 3, Fig. 1B). Intolerance of uncertainty, on the other hand, was the strongest predictor of pandemic-related anxiety in both countries (Table 3). Higher levels of uncertainty intolerance were associated with higher pandemic anxiety scores (Fig. 1C). Moreover, we predicted mindfulness score to be negatively associated with anxiety. This was the case in both countries. People with higher mindful attention awareness score reported lower levels of pandemic anxiety (Table 3, Fig. 1D). Another theoretically relevant co-variate in our study was the perceived risk of catching the novel coronavirus. As predicted (hypothesis 3), people with higher risk perception reported higher levels of pandemic anxiety (Fig. 1E), and risk perception had a significant positive association with pandemic anxiety in both countries (Table 3).

**Table 3.** Multiple regression models of pandemic anxiety and risk avoidance behaviours in the UK and Turkey

	Pandemic-related anxiety						Risk avoidance behaviours					
	UK			Turkey			UK			Turkey		
	*β*	*SE*	*P*	*β*	*SE*	*P*	*β*	*SE*	*P*	*β*	*SE*	*P*
Pandemic-related anxiety		–			–		0.26	0.04	<0.001	0.25	0.02	<0.001
Future-oriented consideration	0.06	0.02	0.018	0.09	0.01	<0.001	0.03	0.03	0.381	0.04	0.01	0.009
Intolerance of uncertainty	0.24	0.02	<0.001	0.29	0.01	<0.001	−0.10	0.03	0.008	−0.07	0.01	<0.001
Mindful Attention Awareness Scale	−0.21	0.02	<0.001	−0.10	0.01	<0.001	0.08	0.03	0.019	0.03	0.01	0.051
Risk perception	0.14	0.00	<0.001	0.16	0.00	<0.001	0.02	0.00	0.488	−0.06	0.00	<0.001
Financial satisfaction	−0.24	0.00	<0.001	−0.15	0.00	<0.001	−0.07	0.00	0.020	−0.02	0.00	0.235
Age	0.04	0.00	0.203	−0.09	0.00	<0.001	0.12	0.00	<0.001	0.11	0.00	<0.001
Sex (female = 0, male = 1)	−0.10	0.04	<0.001	−0.16	0.02	<0.001	−0.07	0.06	0.023	−0.13	0.02	<0.001
Sex (female = 0, other = 1)	−0.01	0.10	0.803	−0.01	0.10	0.698	−0.04	0.15	0.221	−0.02	0.10	0.153
Education (below UG = 0, UG = 1)	−0.12	0.05	<0.001	0.04	0.02	0.029	−0.15	0.06	<0.001	0.06	0.02	0.002
Education (below UG = 0, PG = 1)	−0.15	0.05	<0.001	−0.00	0.03	0.867	−0.15	0.07	<0.001	0.03	0.03	0.113
*F*	51.066***			159.320***			12.524***			33.796***		
*R* ^2^	0.32			0.29			0.11			0.09		

*** *P* < 0.001.

Among our control variables, financial satisfaction had a large effect on pandemic anxiety in both countries. As people’s satisfaction with their financial status increased, their pandemic anxiety decreased (Table 3). Another control variable that was significantly associated with anxiety was sex. In both countries, women reported higher pandemic anxiety than men (Table 3). Pandemic anxiety decreased with increasing age in Turkey, but not in the UK (Table 3). Age did not predict anxiety in the UK. The level of education was negatively associated with anxiety levels in the UK, but not in Turkey (Table 3).

### Pandemic anxiety promoted risk avoidance behaviour

One of our main hypotheses was that people with pandemic anxiety would engage in risk avoidance behaviours more frequently (hypothesis 4). Our results confirmed this hypothesis: people with higher levels of pandemic anxiety reported engaging in risk avoidance behaviours more frequently (Table 3, Fig. 1F). The full models explained the 11% and 9% of the variance in risk avoidance behaviours in the UK and Turkey, respectively. Pandemic anxiety accounted for 5% of this variance in the UK and 4% in Turkey. We further examined bivariate relationships between anxiety variables (i.e. generalized and pandemic anxiety scores) and each of the items on our risk avoidance behaviour scale. All associations were positive and pandemic anxiety had stronger associations with each of the risk avoidance behaviours compared to generalized anxiety in both countries (Table 4). Therefore, pandemic-related worries, more so than general anxiety, contributed to increased engagement with risk avoidance behaviours.

**Table 4.** Pearson correlation coefficients between anxiety variables and risk avoidance behaviours

	UK		Turkey	
Risk avoidance behaviours	Generalized anxiety	Pandemic anxiety	Generalized anxiety	Pandemic anxiety

Stopping attending social gatherings	0.09^**^	0.18^***^	0.01	0.16^***^
Physical distancing	0.04	0.13^***^	0.01	0.14^***^
Staying at home	0.10^**^	0.18^***^	0.05^**^	0.16^***^
Hand washing	0.10^**^	0.21^***^	0.06^***^	0.20^***^
Mask wearing	0.13^***^	0.18^***^	0.03	0.12^***^
Avoiding crowded places	0.10^**^	0.18^***^	0.02	0.16^***^

** *P* < 0.01,

*** *P* < 0.001.

Among control variables, age was a significant predictor of risk avoidance behaviours. In both countries, engagement in risk avoidance behaviours increased with increasing age (Table 3). Moreover, women engaged in risk avoidance behaviours more than men in both countries (Table 3). There was a strong negative association between the level of education and risk avoidance behaviour in the UK (Table 3). In Turkey, this association was positive; however, the effect sizes were small (Table 3). Other variables had either minor or non-significant effects on risk avoidance behaviours (Table 3).

### Participants in Turkey reported higher anxiety and risk avoidance behaviours than those in the UK

The average total GAD-7 score (on a scale of 0–21) was significantly higher in Turkey (*M* = 7.95, *SD* = 5.05) than in the UK (*M* = 6.01, *SD* = 5.12, *t*(1714) = 11.1, *P <* 0.001). Likewise, the average total pandemic-related anxiety score (on a scale of 0–18) was significantly higher in Turkey (*M* = 11.37, *SD* = 4.05) than in the UK (*M* = 8.13, *SD* = 4.08, *t*(1725) = 23.2, *P <* 0.001). The average risk avoidance behaviour score was also significantly higher among the participants in Turkey (3.46 vs 2.56 on a scale of 1–4, *t*(1401) = 32.9, *P <* 0.001).

The mean levels of several correlates of pandemic anxiety also differed between the UK and Turkey. For example, the mean intolerance of uncertainty score was higher in Turkey than in the UK (Table 1, *t*(1623) = 15.9, *P <* 0.001). The average mindful attention awareness score, on the other hand, was higher among the participants in the UK than in Turkey (Table 1, *t*(1647) = −11.0, *P <* 0.001). There was a small but significant difference in future-oriented consideration between the two countries with participants in the UK scoring higher on future consideration (Table 1, *t*(1542) = −7.3, *P <* 0.001). Finally, the average financial satisfaction score was higher in the UK than in Turkey (67 vs 48 on a scale of 0–100, *t*(1930) = −22.3, *P <* 0.001).

## DISCUSSION

In this article, we examined the correlates and adaptive function of anxiety during the first wave of the COVID-19 pandemic in the UK and Turkey. As predicted, people who scored high on generalized anxiety also scored high on pandemic-related anxiety. Our hypothesis 2 concerned the effects of time perspectives (future orientation and mindful attention awareness) and uncertainty intolerance on anxiety levels. In line with our predictions, more future-oriented participants had higher levels of pandemic-related worries; however, the effect sizes were small. The strongest predictor of pandemic anxiety in both countries was intolerance of uncertainty. As predicted, participants with increased mindful attention awareness had lower levels of pandemic anxiety. Perceived risk of catching the virus was positively associated with pandemic anxiety, confirming our hypothesis 3. We found that participants with elevated pandemic-related anxiety levels engaged in risk avoidance behaviour more frequently suggesting that anxiety can help to reduce mortality risk. Finally, generalized and pandemic-driven anxiety levels were higher among Turkish participants whose risk avoidance behaviour scores were also higher than the participants in the UK. Below, we discuss each of these findings.

### Correlates of pandemic anxiety: time perspectives and uncertainty intolerance

We hypothesized that ‘too much’ future orientation in modern societies may be contributing to the recent rise in anxiety disorders, as anxiety at it is core is an emotional response triggered in anticipation of possible future outcomes. Previous research on anxiety showed that anxious individuals exhibit a cognitive bias that they are more likely to attribute negative outcomes to uncertain situations [15, 32] and find it hard to tolerate or accept uncertainty [33, 34]. Moreover, self-labelled worriers are primarily concerned about the uncertain future [13]. Our findings support these observations suggesting that it is not future-oriented thinking per se but intolerance of future uncertainties that contribute to increased anxiety response. Because pandemics such as the COVID-19 bring about many future uncertainties, those who are less tolerant of uncertainty exhibit the highest anxiety response.

Participants with higher mindful attention awareness scores had lower levels of pandemic anxiety. Interestingly, there was a strong negative correlation between mindfulness and intolerance of uncertainty in both countries (Table 2, *r* = −0.53 and −0.43 for the UK and Turkey, respectively). We suspect anxiety, intolerance of uncertainty and mindful attention awareness are indicators of the same psychological construct. It is possible that people who are unable to tolerate uncertainty cannot focus on the present moment because they often engage in mental problem-solving in anticipation of negative future outcomes. This explains the strong relationship between intolerance of uncertainty and mindfulness. Future studies should address the causal relationship between these two concepts. This may also help us better predict the effectiveness of mindfulness-based therapies. Studies have reported positive outcomes of cognitive therapies that involve mindfulness techniques for reducing anxiety [35, 36]; however, others have criticized poor study designs and the lack of universally accepted definition of mindfulness [37]. Training the mind to focus on the present-moment experience may alleviate anxiety, possibly by taking one’s focus away from the future and potential negative outcomes; however, it may not be possible for everyone to simply focus on the present moment, especially if they are highly intolerant of uncertainties.

### Adaptive function of anxiety during a pandemic

Only a few studies have demonstrated the benefits of anxiety [24, 25]; however, an emphasis on ‘diagonal psychology’ (i.e. the benefits of negative states and disadvantages of positive states) can help with better clinical decisions on when to act on emotional states and when a response can be considered normal [6]. We found a strong correlation between an individual’s overall anxiety level (measured as generalized anxiety) and their level of pandemic-related worries, such as feeling stressed about leaving their house or being worried about their/their family’s health. Because individuals with high anxiety are predicted to have lower threat detection threshold, we predict their pandemic-related anxiety to also be higher. Furthermore, the anxiety subtypes (e.g. various anxiety disorders) can be considered as partially differentiated responses of a general anxiety response adapted to different threat situations [23]. For example, while social threats may trigger an anxiety response that evokes submissive behaviour, an encounter with a predator may trigger a response promoting freezing behaviour [23]. Following this, pandemic-driven anxieties are expected to trigger avoidance behaviours to protect against infectious agents. Although our regression models did not explain a large proportion of the variance in risk avoidance behaviours, among all covariates pandemic anxiety explained the largest variation in both countries.

The association between pandemic anxiety and risk avoidance behaviours found in this study suggests that inducing anxiety may be an effective public health intervention to increase protective behaviours during pandemics. Because anxiety is a response expressed in anticipation of threats, clear communication of risk of disease can promote protective behaviour. A recent study has shown that higher perceived risk of infection increased self-reported engagement in protective behaviours during the first week of the COVID-19 pandemic in the USA [38]. In our study, when considered together with anxiety, the perceived risk of catching the virus did not predict engagement in risk avoidance behaviours in the UK, and only had a minor effect in Turkey. Therefore, we believe that the association between risk perception and engagement in protective behaviours is driven by the anxiety response. In addition, we have shown elsewhere that participants with higher pandemic-related anxieties were more likely to vaccinate against COVID-19 [26]. Nevertheless, it is important to note that we cannot say with certainty that the observed association between anxiety and protective behaviours in this study is causal.

Our findings bring about the question of what level of anxiety can be considered normal and the cost of being overly anxious. In clinical psychology, a condition is thought to be pathological if it is impairing the quality of life of an individual. An evolutionary perspective suggests that if a biological system is not producing the effects that it was selected for and is leading to harm, then it is not functioning normally and can be considered a disorder [39]. In the case of anxiety, a decision on whether to intervene with a therapeutic method can be based on asking whether the individual is avoiding situations and activities that are harmless or even beneficial. It is important to acknowledge here that the costs and benefits associated with the anxiety response are context dependent. During pandemics, avoiding risk can be costly as it can lead to a loss of livelihoods. For example, people of lower socioeconomic status may not be able to engage in risk avoidance behaviour in fear of losing jobs and thus face increased risk of infection [40]. On the other hand, an increased anxiety response may benefit certain individuals more so than others. As part of the *behavioural immune system* individuals who are vulnerable to infection are predicted to elicit more aversive responses [41]. For example, the benefit of an anxiety response during a pandemic will be larger for an individual with immune deficiency.

### Demographic correlates of pandemic anxiety and risk avoidance behaviours

Among our control variables, financial satisfaction was the strongest correlate of pandemic anxiety. It is not surprising that participants who were less satisfied with their financial status had higher levels of pandemic-related anxiety given that the pandemic resulted in job insecurities and potential financial loss. Another demographic variable that was significantly correlated with pandemic anxiety was sex. Women in both countries reported experiencing higher levels of pandemic anxiety than men. This result is consistent with previous studies showing that women experience anxiety more and are twice more likely to develop anxiety disorders over their lifetime than men [42]. Women also reported engaging in risk avoidance behaviours more often than men in both countries. It may be that as women experience higher pandemic anxiety, they also take more caution and engage in protective behaviours. Interestingly in the UK, participants with undergraduate and postgraduate degrees reported less pandemic anxiety compared to those without a university degree. It is possible that their reduced anxiety contributed to the lesser engagement in risk avoidance behaviours reported by these participants. Finally, although older participants did not report higher levels of anxiety, they engaged in risk avoidance behaviours to a greater extent compared to younger participants in both countries. It is possible that as people get older, they may get more experienced at coping with uncertainties, which render them less susceptible to anxiety. Indeed, we found a strong significant negative correlation between age and uncertainty intolerance in both countries (*r* = −0.31 for UK and *r* = −0.22 for Turkey). These findings suggest that despite high mortality risk for the elderly during the COVID-19 pandemic, their increased tolerance of uncertainty results in decreased anxiety levels.

### Country-level differences

We found differences in the overall emotional and behavioural response to the pandemic between the UK and Turkey. For example, both generalized anxiety levels and pandemic-related anxiety levels were higher among Turkish participants. There was a significant difference in the mean intolerance of uncertainty score between the two countries (on a scale of 1–5, *M*_Turkey_ = 3.49 vs *M*_UK_ = 2.94), which was probably the main driver behind the higher anxiety scores in Turkey. Another factor that potentially contributed to the elevated levels of pandemic anxieties in Turkey was financial satisfaction. The average level of financial satisfaction, on a scale of 0–100, was 48 for Turkish participants and 67 for the participants in the UK. Engagement in risk avoidance behaviour during the first wave of the pandemic was also significantly higher among the Turkish participants. It is possible that elevated anxiety levels in Turkey rendered people to take more precautions. The difference in protective behaviour may also be due to the cultural differences in collectivist attitude (individualism score for Turkey is 37, as opposed to 89 for the UK) [43]. Indeed, levels of collectivism were associated with higher intentions to engage in social distancing behaviours and mask wearing during the COVID-19 pandemic [44, 45]. Our findings showed that there was an especially large difference in mask wearing behaviour between the two countries (Table 1).

There are limitations to our study that should be considered. First, our sample was composed of voluntary participants who were likely interested in behavioural aspects of COVID-19, therefore may not be random. The overall level of education was higher among our participants compared to the population-specific education levels. Second, over 60% of our participants in both countries were women. Therefore, overall mean generalized and pandemic anxiety levels found in this study should be interpreted carefully, as women report experiencing higher anxiety than men. Likewise, the overall reported engagement in risk avoidance behaviours may be higher in our sample in both countries as women reported engaging in these behaviours more frequently. Finally, our study did not measure the actual mortality from COVID-19 but used risk avoidance behaviour as an indirect measure for mortality risk.

## CONCLUSIONS AND IMPLICATIONS

Our study shows that an elevated anxiety response can be beneficial in avoiding risk of infection during pandemics. Country-level differences in engagement with protective behaviours during a pandemic may be driven by differences in overall anxiety levels. These findings open further discussions on the normal anxiety response and stress the importance of the context in which an anxiety response is triggered. Our findings also add to the growing discussions on mindfulness-based therapies, showing that mindfulness is highly correlated with uncertainty intolerance—the largest predictor of anxiety. Therapies that focus on being more tolerant of uncertainties can alleviate anxiety. Finally, maintaining a healthy level of anxiety during a pandemic can promote protective behaviours.

## Supplementary Material

eoab037_Supplementary_Data
