# Supplementary material for: Adaptive function and correlates of anxiety during a pandemic
Source: Evol Med Public Health. 2021 Nov 12;9(1):393–405. doi: 10.1093/emph/eoab037 (PMC8651168; doi:10.1093/emph/eoab037)
Supplement: eoab037_Supplementary_Data [file eoab037_supplementary_data.docx]

**Supplementary Information**

**Adaptive function and correlates of anxiety during a pandemic**

Gul Deniz Salali^1^, Mete Sefa Uysal^2^, Abi Bevan^1^

^1^Department of Anthropology, University College London, London WC1H 0BW, UK

^2^Department of Psychology, Dokuz Eylül University, Izmir, Turkey

*Corresponding author: Gul Deniz Salali

**Email:**  [guldeniz.salali@ucl.ac.uk](mailto:guldeniz.salali@ucl.ac.uk)

| **Supplementary Table 1**. Demographic Information | | |
| --- | --- | --- |
|  | **Mean (SD)/n(%)  UK** | **Mean (SD)/n(%)  Turkey** |
| Age (years) | 44.33 (13.70) | 31.88 (11.22) |
| Gender |  |  |
| Female | 735 (67.6%) | 2445(62.1%) |
| Male | 322 (29.6%) | 1457 (37.0%) |
| Other | 31 (2.8%) | 33 (0.9%) |
| Education |  |  |
| Below Graduate | 265 (24.4%) | 1130 (28.7%) |
| Graduate Degree | 442 (40.6%) | 2117 (53.8%) |
| Postgraduate Degree | 381 (35.0%) | 688 (17.5%) |
| Financial Satisfaction (0-100) | 67.32 (24.91) | 47.71 (28.25) |
| Children |  |  |
| Yes | 559 (51.4%) | 976 (24.8%) |
| No | 529 (48.6%) | 2959 (75.2%) |
| Ethnicity |  |  |
| White | 949 (87.2%) | - |
| Other | 139 (12.8 %) | - |
| Live with |  |  |
| Alone | 151 (13.9%) | 424 (10.8%) |
| Other people (friend(s) or housemate(s) but not family) | 103 (9.5%) | 198 (5.0%) |
| Family (a partner, or partner and children, or other family members) | 829 (76.2%) | 3236 (82.2%) |
| Other | 5 (0.5%) | 77 (2.0%) |
| Region UK |  |  |
| South England | 821 (75.5%) | - |
| Midlands of England | 74 (6.8%) | - |
| North England | 136 (12.5%) | - |
| Scotland, Northern Ireland, Wales | 57 (5.2%) | - |
| Region TR |  |  |
| Istanbul | - | 1373 (34.9%) |
| Ankara | - | 526 (13.4%) |
| Izmir | - | 514 (13.1%) |
| Other | - | 1522 (38.7%) |
